# Walking Corpse Syndrome: A Case Report of Cotard's Syndrome

**DOI:** 10.7759/cureus.63824

**Published:** 2024-07-04

**Authors:** Karlyle Bistas, Maheen Mirza

**Affiliations:** 1 Behavioral Health, Wayne State University/Detroit Medical Center, Detroit, USA; 2 School of Medicine, Medical University of the Americas, Charlestown, KNA

**Keywords:** cotard’s syndrome, delusions, walking corpse syndrome, delusions of negation, nihilistic delusions

## Abstract

Nihilistic delusions are unique psychopathological experiences characterized by the belief of being deceased, decayed, or obliterated. This case report sheds light on a patient grappling with nihilistic delusions, highlighting the strategies for treating and managing this psychiatric condition. The pathophysiology of Cotard's syndrome remains elusive, with proposed mechanisms being largely speculative. Further research is imperative to gain a comprehensive understanding of the underlying mechanisms. Neurological assessments should be conducted in patients with Cotard's syndrome to rule out organic etiologies.

## Introduction

Nihilistic delusions, also known as délires de négation (delusions of negation), are unique psychopathological experiences marked by an unwavering belief in being deceased, decomposed, or annihilated [1]. Individuals grappling with these delusions may perceive a loss of internal organs or question their existence [1]. While infrequent, nihilistic delusions can occur within the spectrum of schizophrenia. This syndrome is also termed corpse syndrome, wherein affected individuals believe they are already deceased, non-existent, or in a state of putrefaction [2].

Cotard delusion encompasses not only the sensation of being dead or decaying but also manifests as anxiety, depression, agitation, suicidal ideation, and other delusional beliefs [3]. First described by Dr. Jules Cotard in 1882, Cotard's syndrome is a relatively rare condition observed in patients with mood disorders, psychotic disorders, central nervous system (CNS) infections, CNS tumors, and traumatic brain injuries [2]. This neuropsychiatric condition presents with psychotic depression characterized by anxiety, delusions of guilt, depression, and auditory hallucinations. Cotard's syndrome type I features hypochondriacal and nihilistic delusions regarding the body and existence, and Cotard's syndrome type II features anxiety, delusions of immortality, auditory hallucinations, nihilistic delusions regarding existence, and suicidal behaviors [3,4-5].

Cotard's syndrome can be conceptualized along a spectrum with varying degrees of symptom severity. Yamada and colleagues delineated three distinct stages: the germination stage (associated with prodromal depression and hypochondriacal symptoms), the blooming stage (full development of the syndrome with delusions of negation), and the chronic stage (chronic depressive or delusional type) [6]. This case report highlights a patient experiencing nihilistic delusions linked to schizophrenia. During an acute episode, the patient presented to the emergency department, firmly convinced of his death. The report explores the presentation, pathophysiology, and treatment of this syndrome.

## Case presentation

Law enforcement brought a 60-year-old male, with schizophrenia and polysubstance abuse, to the hospital due to aggressive behavior. He expressed paranoid and nihilistic thoughts and delusions. Collateral information revealed medication non-compliance and threatening behavior. He expressed feeling as though "people are out there murdering everybody" and distressing thoughts such as "How can I calm down when I am already dead?" His homeowner petitioned him for throwing an item out of his apartment window, bizarre behavior, and medication non-adherence, leading him to the Emergency Department (ED) to be examined, where the interview ended early due to erratic behavior. The patient was screaming, thrashing, and lunging toward the writer.

During the assessment, the patient appeared disheveled. He was agitated, displaying disorganized speech and thought patterns indicative of psychosis, including delusions and auditory hallucinations. He looked his age, restrained on the ED bed, holding a religious text. His speech was disorganized with increased volume and irritability, and he exhibited psychomotor agitation. He reported his mood as "I am dead," with a dysphoric affect, intense eye contact, and religious preoccupation. His thought process was coherent but disorganized, with poor insight and judgment. He showed nihilistic, paranoid, and persecutory delusions, along with auditory and visual hallucinations, responding to internal stimuli. Despite initial tachycardia, his vital signs were normal, and physical examination was unremarkable. Laboratory tests indicated positive results for benzodiazepines and cannabis. The patient was deemed acutely psychotic and a danger to himself and others, necessitating inpatient admission. He was at a high risk of harm to himself and others due to not being reality-based and displaying violent behavior. A petition and clinical certification were completed. He was started on risperidone 2 mg twice daily for psychosis and benztropine 2 mg daily for extrapyramidal symptoms in the ED.

## Discussion

Signs and symptoms 

Cotard's syndrome, also recognized as Cotard delusion or walking corpse syndrome, is a rare psychiatric disorder characterized by the conviction that one is deceased or has lost vital organs. This condition encompasses delusional beliefs, self-denial, depressive manifestations, social withdrawal, and detachment from self. The central feature of this syndrome lies in the delusional convictions surrounding one's mortality or non-existence. Patients may reject the presence of their bodily organs and assert that they have vanished. Moreover, they may perceive sensations of decay or decomposition and feel disconnected from their being. Such distorted perceptions often lead to social isolation and depressive states. In addition, hallucinations may manifest, particularly as auditory or olfactory hallucinations. The declaration "I am already dead" stands as a characteristic symptom of Cotard's syndrome, denoted as nihilistic delusions [7].

Pathophysiology

Understanding the pathophysiology of Cotard's syndrome remains elusive due to its rarity, hindering comprehensive research efforts. Some studies suggest a link between Cotard’s syndrome and structural brain abnormalities, alongside functional irregularities [5]. These changes are thought to be localized in the frontal and temporal lobes, areas associated with self-awareness and reality processing [5]. In addition, Cotard’s syndrome often accompanies nihilistic delusions and other psychiatric conditions.

Treatment

Electroconvulsive therapy (ECT) emerges as a viable treatment option for Cotard’s syndrome. Single-photon emission computed tomography (SPECT) scans conducted immediately post-ECT and after one month revealed enhanced perfusion in specific brain regions, including the left temporal lobe, left inferior front, and left parietal lobe [6].

While pharmacotherapy proves effective in about half of Cotard’s syndrome cases, modified electroconvulsive therapy (mECT) may be necessary to alleviate symptoms. Antipsychotics, notably clozapine, have been explored for Cotard’s syndrome treatment. In one case, clozapine administration over three months resulted in a bilateral decrease in D2 receptor binding, as observed through imaging [8].

Research indicates promising outcomes with paroxetine administration for 36 days, supplemented by pramipexole starting from day 37, leading to significant improvement and complete resolution within 15 days [9]. In cases of paranoid schizophrenia, studies demonstrate the efficacy of paliperidone at 12 milligrams per day alongside lorazepam at 5 milligrams per day [10].

For individuals grappling with Cotard’s syndrome and schizophrenia, aripiprazole at 30 milligrams per day in conjunction with clonazepam at 2 milligrams per day has been beneficial, with substantial improvement observed within two weeks [11]. Moreover, studies reveal the successful management of depressive disorder with psychotic symptoms using imipramine at 150 milligrams per day alongside risperidone at 6 milligrams per day, resulting in complete symptom resolution [12].

Treatment approaches for Cotard's syndrome vary depending on its clinical etiology, often linked to an underlying disorder. Patients receive tailored interventions based on the associated mood disorder, including antipsychotics, antidepressants, and ECT. Available treatments encompass ECT, psychopharmacology, behavioral and psychotherapies, as well as discontinuation of triggering medications. Cotard delusions manifest across a broad spectrum of neurological, psychiatric, and medical conditions, exhibiting diverse neural alterations. Lesions in the nondominant hemisphere may contribute to the syndrome’s pathophysiology, with several effective treatment modalities at hand [8,9].

Neuroanatomical associations

Neuroimaging studies have detected frontal lobe changes [5]. Aradhana and Josephs observed frontal lobe changes in four patients, volume loss in four, ischemic changes in five, and right-sided or bilateral hemisphere lesions in seven [5]. They also suggested that nondominant hemisphere lesions may contribute to the pathophysiology [5].

The literature on Cotard's syndrome includes structural neuroimaging studies revealing bilateral cerebral atrophy, enlargement of the Sylvian and interhemispheric fissures, and dilation of lateral ventricles in patients [6]. Drake's study described structural lesions in the right frontotemporal region and temporal lobe epilepsy in patients with Cotard's syndrome [7]. Limited SPECT studies on Cotard's syndrome cases have shown bilateral hypoperfusion in the dorsolateral frontal lobes, frontoparietal medial cortex, basal ganglia, and thalamus [6].

De Risio investigated D2 receptor binding using SPECT, noting reduced striatal D2 receptor binding with greater uptake on the left side [8]. Brain electrical activity mapping has also been employed in Cotard’s syndrome, revealing electrophysiological abnormalities with a right temporal predominance [9]. Neuropsychological studies have indicated severe impairment in facial recognition tasks, including the Warrington Recognition Memory Test, recognition of emotional facial expressions, and familiar faces [6].

Review of the current literature

Cotard's syndrome has been associated with organic conditions, such as temporal lobe epilepsy (TLE), head injury, or brain tumors [6]. Consequently, neuropsychological or neurostructural abnormalities may manifest as symptoms of Cotard’s syndrome. CT/MRI studies have indicated abnormalities in the non-dominant frontal, temporal, and occasionally parietal lobes in Cotard's syndrome cases [6]. Other research has also linked Cotard’s syndrome to abnormalities in the non-dominant cerebral hemisphere [9].

The literature suggests that Cotard’s syndrome is linked to dysfunction of "interoception," which involves internal bodily sensations [10]. This dysfunction may impact emotional development, as abnormal interceptive processing could lead to a loss of emotional resonance or feelings of emptiness [10]. The two-factor theory of delusions proposes that the development of delusions requires both perceptual experience and abnormal reasoning regarding those perceptions [10]. Impaired interoception could disrupt emotional engagement during social interactions, potentially leading to nihilistic delusions [10].

Studies have also explored the occurrence of Cotard’s syndrome in severe depression. Untreated patients with depression may develop nihilistic delusions over time [13,14]. A three-stage model of disease progression has been proposed, beginning with a gradual depressive state, progressing to nihilistic delusions, and culminating in a chronic stage with persistent mood changes and delusions [15].

To further understand the etiology of Cotard’s syndrome, neuroimaging has been utilized to assess the extent of damage. Right-sided and bilateral cerebral atrophy, typically affecting the frontal lobe, has been frequently observed [5,16]. Given the frontal lobe's involvement in various aspects of mental and cognitive functioning, damage to this region can disrupt neural circuits, leading to abnormal perceptions of self [16]. In addition, damage to neural circuits can interfere with connections between the temporal lobe and limbic system, resulting in misunderstandings of self, people, places, and objects, which may explain the presence of nihilistic delusions in this syndrome [16].

Associations with other mental illnesses 

Cotard’s syndrome is predominantly associated with psychoses like schizophrenia and bipolar disorder [2]. However, it has also been documented in cases of organic lesions affecting the nondominant temporoparietal cortex, as well as in individuals experiencing migraines [2]. It tends to affect women more frequently and is commonly observed in middle-aged to older patients [10]. This syndrome has been reported in patients with a range of psychiatric and neurological conditions, including schizophrenia and affective disorders [10]. In the case at hand, the patient was diagnosed with schizoaffective disorder.

## Conclusions

This case report delves into the rare psychiatric phenomenon known as Cotard’s syndrome, highlighting the importance of prompt intervention. Further exploration of the underlying pathophysiology of Cotard’s syndrome is crucial for discovering additional treatment options and approaches. Specific neuroanatomical associations with this phenomenon have been observed, suggesting potential avenues for targeted treatment. Understanding the etiology of the delusions is vital, as it dictates the appropriate treatment approach.
